# The effect of cognitive rehabilitation on daily functioning of patients with Alzheimer’s disease: a systematic review and meta-analysis of clinical trials

**DOI:** 10.3389/fneur.2024.1371298

**Published:** 2024-04-02

**Authors:** Shuyu Ren, Fangfang Pan, Jie Jin

**Affiliations:** Rehabilitation Department, Linping Campus, Second Affiliated Hospital, Zhejiang University School of Medicine, Hangzhou, China

**Keywords:** Alzheimer disease, cognitive rehabilitation, functional cognitive domains, meta-analysis, system assessment

## Abstract

**Background:**

Alzheimer’s disease (AD) is the most prevalent type of dementia and represents 60–80% of dementia cases. AD affects over 32 million people globally, and 8.1% of affected females and 5.4% of affected males were older than 65 years. Cognitive rehabilitation focuses on helping patients develop individualized strategies to obtain or maintain optimal functioning. As of now, there is no complete and systematic meta-analysis on the effects of cognitive rehabilitation on cognitive functioning in AD patients.

**Objectives:**

To provide the most recent and extensive pooled analysis and evidence and explore the influence of cognitive rehabilitation on overall cognitive functioning in patients with AD.

**Methods:**

We searched articles through several databases such as PubMed, Cochrane Library, Embase, and Web of Science, from the inception to June 2023. Studies on cognitive stimulation, cognitive training, and cognitive interventions, and non-English articles were excluded. The outcome measures encompassed the effects of cognitive rehabilitation on the overall cognitive functioning of people with AD (e.g., verbal fluency, behavioral memory, neuropsychiatric status and occupational performance levels).

**Results:**

A total of 14 clinical trials were included in this analysis. The meta-analysis showed that cognitive rehabilitation significantly improved quality of life (WMD: 2.87; 95% CI: 0.79, 4.95; *p* = 0.007) and occupational performance levels (WMD: 1.53; 95% CI: 0.43, 2.63; *p* = 0.007) in patients with AD. However, it did not show a significant effect on other domains of specific cognitive functions in patients with AD.

**Conclusion:**

Cognitive rehabilitation exhibited a moderate to large impact on both quality of life and occupational performance levels in people with AD. Future studies are required to explore the potential of various cognitive interventions across specific domains, so as to provide more insights into the management of AD.

**Systematic review registration:**

https://www.crd.york.ac.uk/prospero/, identifier CRD42023444390.

## 1 Introduction

Dementia is characterized by cognitive functioning and behavior impairments and affects over 55 million people globally. In 2040, the number of people with dementia is projected to be 90 million (1). Alzheimer’s disease (AD) is the most prevalent type of dementia and represents 60–80% of dementia cases (2). AD affects over 32 million people globally (3), and 8.1% of affected females and 5.4% of affected males were older than 65 years. AD is an irreversible and degenerative disease that causes a gradual decline in memory and thinking, resulting in difficulty in performing basic daily activities. In the pre-AD stage, an individual can typically perform most daily tasks with minimal difficulty. As the disease progresses, various cognitive areas, including semantic, practical, and executive function, are impaired, resulting in decreased situational memory, behavioral changes, and impaired verbal and visuospatial abilities (4). In the later stages, severe cognitive and functional limitations emerge, often accompanied by behavioral changes such as apathy, depression, aggression, and agitation.

Current pharmacotherapy interventions for AD primarily target symptoms without altering the course of the disease (5). Additionally, non-pharmacologic interventions that improve or maintain cognitive function offer promise in helping individuals with AD and their caregivers (6). Interventions such as cognitive stimulation, cognitive training, and cognitive rehabilitation are actively employed. Cognitive stimulation aims to promote socialization activities and group discussions, which focuses on increasing or maintaining cognitive and social functioning in specific domains (7). Cognitive training involves various tasks tailored to enhance specific cognitive functions based on individual performance (8). Cognitive rehabilitation focuses on enhancing the patient’s capacity to manage daily activities, including learning or relearning important information and maintaining this knowledge over time, with guidance from family members and/or healthcare professionals. These endeavors help patients develop individualized strategies to obtain or maintain optimal functioning (9).

It is worth noting that outside the search window of our study, a latest meta-analysis, titled *cognitive rehabilitation for people with mild to moderate dementia*, was published by Kudlicka et al. in 2023. Their study included 6 eligible randomized controlled trials published in English from 2010 to 2022, involving a total of 1,702 subjects. They found that CR has a large positive impact on three main outcomes: self-rating of performance, informant ratings of goal attainment, and self-ratings of satisfaction with goal attainment. However, unlike the study by Kudlicka, our study focuses on AD patients. Furthermore, the meta-analysis by Kudlicka only includes RCT trials, whereas our study includes 2 additional non-RCT trials, which can provide more comprehensive data.

Despite the large number of separate studies conducted in this area, as of now, there is no complete and systematic meta-analysis on the effects of cognitive rehabilitation on cognitive functioning in AD patients. Therefore, this study aims to quantitatively evaluate the overall impact of cognitive rehabilitation on cognitive functioning in AD patients, extending and providing new insights into the management of AD. Through an exhaustive literature review and rigorous methodology, we explore the impact of cognitive rehabilitation on overall cognitive functioning, verbal fluency, behavioral memory, neuropsychiatric status, the ability to perform daily activities, quality of life, and occupational performance levels of AD patients.

## 2 Materials and methods

### 2.1 Literature search

This study was conducted following the PRISMA (Preferred Reporting Items for Systematic Reviews and Meta-Analyses) 2020 statement (10) and prospectively registered in **PROSPERO (CRD42023444390)**. The PRISMA 2020 checklist is presented in Appendix 1.

We conducted a comprehensive literature search up to June 2023 to collect studies that compare the effectiveness of cognitive rehabilitation and alternative interventions for Alzheimer’s disease. The search used several databases such as PubMed, Cochrane Library, Embase, and Web of Science, and all selected articles were in English language. We used the following terms to obtain relevant studies: “Alzheimer Disease,” “Alzheimer Dementia,” “Dementia, Senile,” “Alzheimer Type Dementia,” “Primary Senile Degenerative Dementia,” “Alzheimer Sclerosis,” “Alzheimer Syndrome,” cognitive rehabilitation,” etc. The detailed search strategy is provided in Appendix 2. We manually examined the reference lists of all eligible studies. Two researchers independently assessed the included studies, and any disagreements during article screening were resolved by discussion.

### 2.2 Eligibility criteria

Inclusion criteria for the selected studies were as follows: (1) the study design was a randomized or non-randomized controlled design, (2) the study involved patients diagnosed with AD, and (3) the study compared cognitive rehabilitation with other cognitive interventions; (4) at least one of the following assessment tools was employed: Mini-Mental State Examination (MMSE), Dementia Quality of Life (DQoL), Neuropsychiatric Inventory severity (NPI severity), Neuropsychiatric Inventory distress (NPI distress), Zarit Burden Interview (ZBI), Activities of daily living (ADL), Bayer Activities of Daily Living (B-ADL), Rivermead Behavioural Memory Test(RBMT), Canadian Occupational Performance Measure (COPM), Trail Marking Test (TMTA), verbal fluency; (5) sufficient data was available to calculate the relative risk (RR) or weighted mean difference (WMD).

Exclusion criteria were as follows: (1) letters, reviews, case reports, editorial comments, conference abstracts, and unpublished articles; (2) non-English articles; (3) studies focusing on cognitive stimulation, cognitive training, and cognitive interventions were also discarded, since cognitive rehabilitation was defined as a personalized approach that requires health professionals, people with acquired cognitive impairment, and their families to jointly determine treatment goals and develop intervention strategies, with the primary goal of maintaining the patient’s ability to participate in meaningful daily activities.

### 2.3 Quality assessment

The quality assessment of eligible clinical trials was conducted following the Cochrane Handbook for Systematic Reviews of Interventions 5.1.0 based on seven domains: random sequence generation, allocation concealment, blinding of participants and personnel, blinding of outcome assessment, incomplete outcome data, selective reporting, and other sources of bias (11). Each domain was rated as having a low, high, or unclear risk of bias. Studies with with an overall low risk of bias were regarded as high-quality.

### 2.4 Data extraction

Data extraction was conducted independently by two researchers. In case of disagreements, a third researcher intervened to facilitate a final decision. The extracted data included the first author, year of publication, study period, country of study, sample size, age, gender, and duration of education, MMSE raw score, MMSE, Dem entia Quality of Life (DQoL), NPI severity, NPI distress, ZBI, ADL, B-ADL, RBMT, COPM, TMTA, and/or verbal fluency. When continuous variables were presented as medians with ranges or interquartile ranges, we calculated the mean ± standard deviation by validated mathematical methods (12, 13). We contacted the corresponding author if data were absent or not disclosed.

### 2.5 Statistical analysis

Data was analyzed by using Review Manager version 5.4.1 (Cochrane Collaboration, Oxford, United Kingdom). WMD and RR were employed to analyze and compare continuous and dichotomous variables, respectively. 95% confidential intervals (CIs) were also provided. Heterogeneity was evaluated by chi-square (*χ*^2^) test (Cochran’s Q) and index of inconsistency (*I*^2^) (14). A high heterogeneity was defined as *χ*^2^ *p*-value <0.05 or *I*^2^ >50%. If significant heterogeneity was detected, a random effects model was applied to estimate the combined WMD or RR. Otherwise, a fixed effects model was employed. In addition, we performed one-way sensitivity analyses to evaluate the effect of an individual study on the pooled results of outcomes with significant heterogeneity. Funnel plots were created by Review Manager version 5.3 (Cochrane Collaboration, Oxford, United Kingdom), and Egger regression tests were carried out using Stata version 12.0 (Stata Corp, College Station, TX, United States) (15) to visually assess the published bias for the outcomes reported in 10 or more included studies. A *p*-value <0.05 was considered statistically significant publication bias.

## 3 Results

### 3.1 Literature search and study characteristics

**Figure 1:**
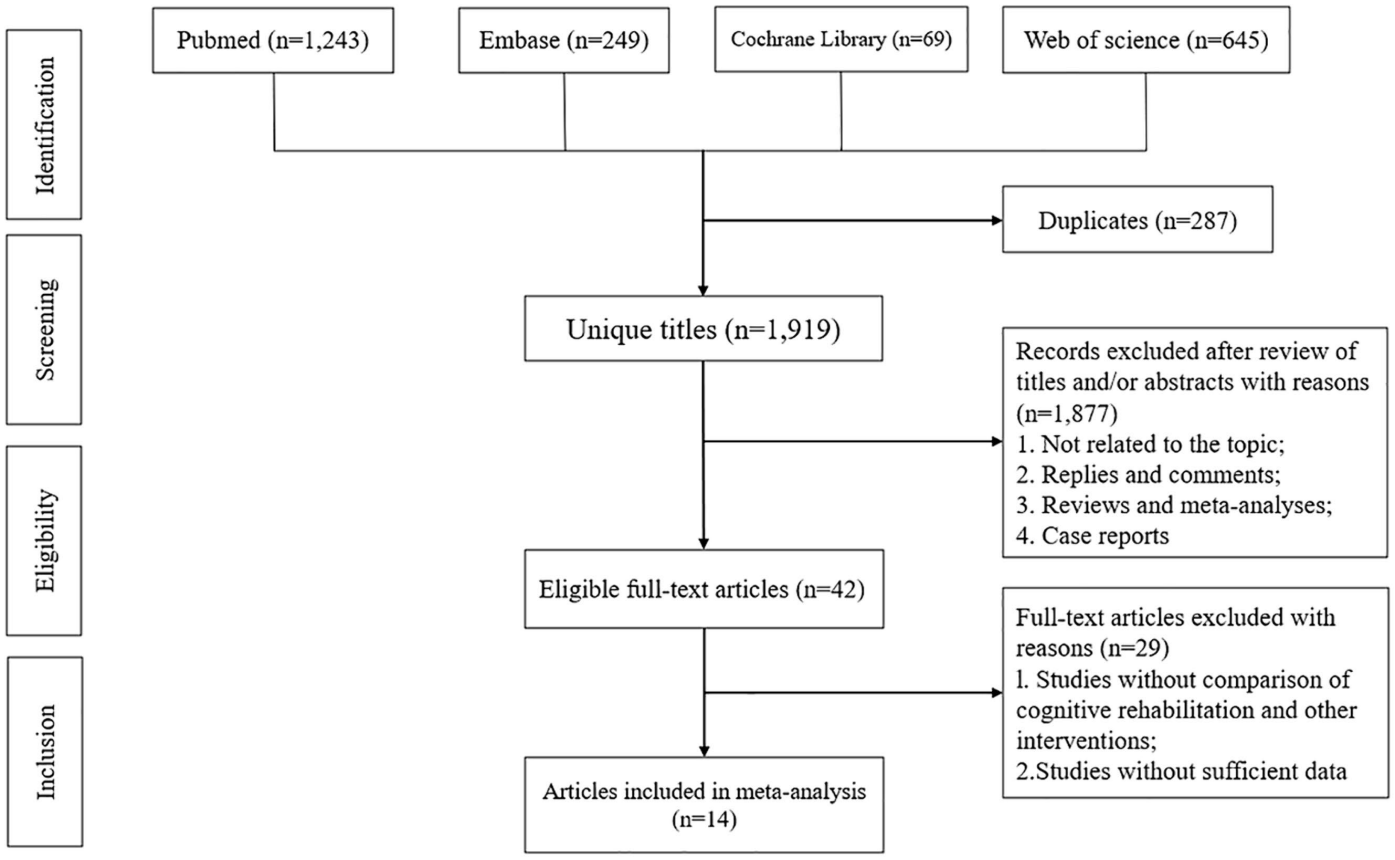
Flowchart of the systematic search and selection process.

The schematic figure of the selection process is illustrated in Figure 1. A total of 2,206 articles were derived from several databases such as PubMed (*n* = 1,243), Embase (*n* = 249), Cochrane Library (*n* = 69), and Web of Science (*n* = 645). After eliminating 287 duplicate articles, 1,919 articles underwent title and abstract screening. Finally, 14 full-text articles involving 1,418 patients (715 patients in the cognitive rehabilitation group and 703 patients in the control group) were included in the pooled analysis (16–29). The characteristics of each study are presented in Table 1.

**Table 1:** Baseline characteristics of include studies and methodological assessment.

Authors	Study period	Country	Study design	Patients (*n*)	Follow-up (months)
				CR/Control	
Amieva et al.	2008–2011	France	RCT	156/168	24
Bottino et al.	2003–2004	Brasil	RCT	6/7	7
Brueggen et al.	2016–2017	Germany	RCT	8/8	14
Clare et al.	2006–2009	UK	RCT	22/24	8
Clare et al.	2013–2016	UK	RCT	239/236	9
Jelcic et al.	2013–2014	Italy	Non-RCT	17/10	3
Kim et al.	2015	Korea	RCT	22/21	2
Kurth et al.	2018–2019	Belgium	Non-RCT	33/17	12
Kurz et al.	2010–2011	Germany	RCT	100/101	9
Laurence et al.	2012–2013	Canada	RCT	7/8	6
Loewenstein et al.	2002–2003	USA	RCT	25/19	7
Paasschen et al.	2013	UK	RCT	7/12	2
Thivierge et al.	2008–2011	Canada	RCT	9/8	6
Zhong et al.	2020–2021	China	RCT	64/64	6

### 3.2 Risk of bias in included studies

**Figure 2:**
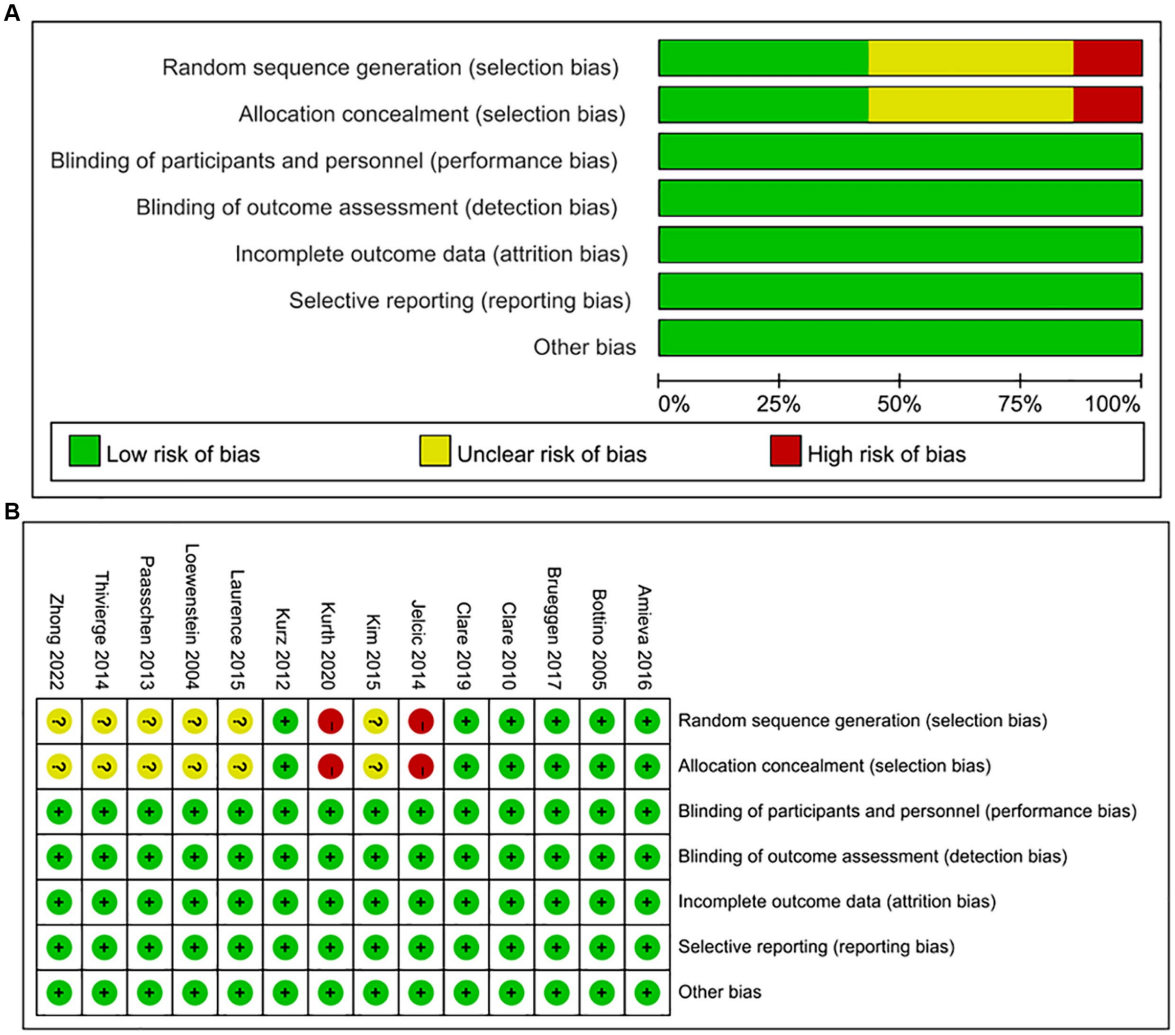
**(A)** Risk of bias graph: risk of bias for each item in the included studies, presented as percentages. **(B)** Risk of bias summary: risk of bias for each item in each included study.

The assessment of bias risk in the included studies is provided in Figure 2A. The risk of bias for specific outcomes in different studies is shown in Figure 2B. A small number of the studies exhibited a high risk of bias in the random sequence generation process. This high risk could be attributed to insufficient data, thus making it challenging to assess the random sequence generation adequately or the attempts to conceal the random sequence.

### 3.3 Demographic characteristics

No significant differences were noted between the two groups in four variables, including age (WMD: −0.34; 95% CI: −1.06, 0.37; *p* = 0.35), sex (men/total, RR: 0.98; 95% CI: 0.88, 1.10; *p* = 0.78), years of education (WMD: −0.03; 95% CI: −0.43, 0.38; *p* = 0.89), and MMSE raw score (WMD: 1.18; 95% CI: −0.12, 2.49; *p* = 0.08) (Table 2).

**Table 2:** Demographics and clinical characteristics of included studies.

Outcomes	Studies	No. of patients	WMD or RR	95% CI	*p*-value	Heterogeneity			
		CR/Control				Chi^2^	df	*p*-value	*I*^2^ (%)
Age (years)	(13)	707/695	−0.34	[−1.06, 0.37]	0.35	13.72	12	0.32	13
Gender (male)	(13)	707/695	0.98	[0.88, 1.10]	0.78	5.53	12	0.94	0
Education (years)	(11)	487/463	−0.03	[−0.43, 0.38]	0.89	10.08	10	0.43	1
MMSE raw score	(12)	643/631	1.18	[−0.12, 2.49]	0.08	154.92	11	<0.00001	93

MMSE, Mini-Mental State Examination; WMD, weighted mean difference; RR, risk ratio; CI, confidence interval.

#### 3.3.1 Change in MMSE

**Figure 3:**
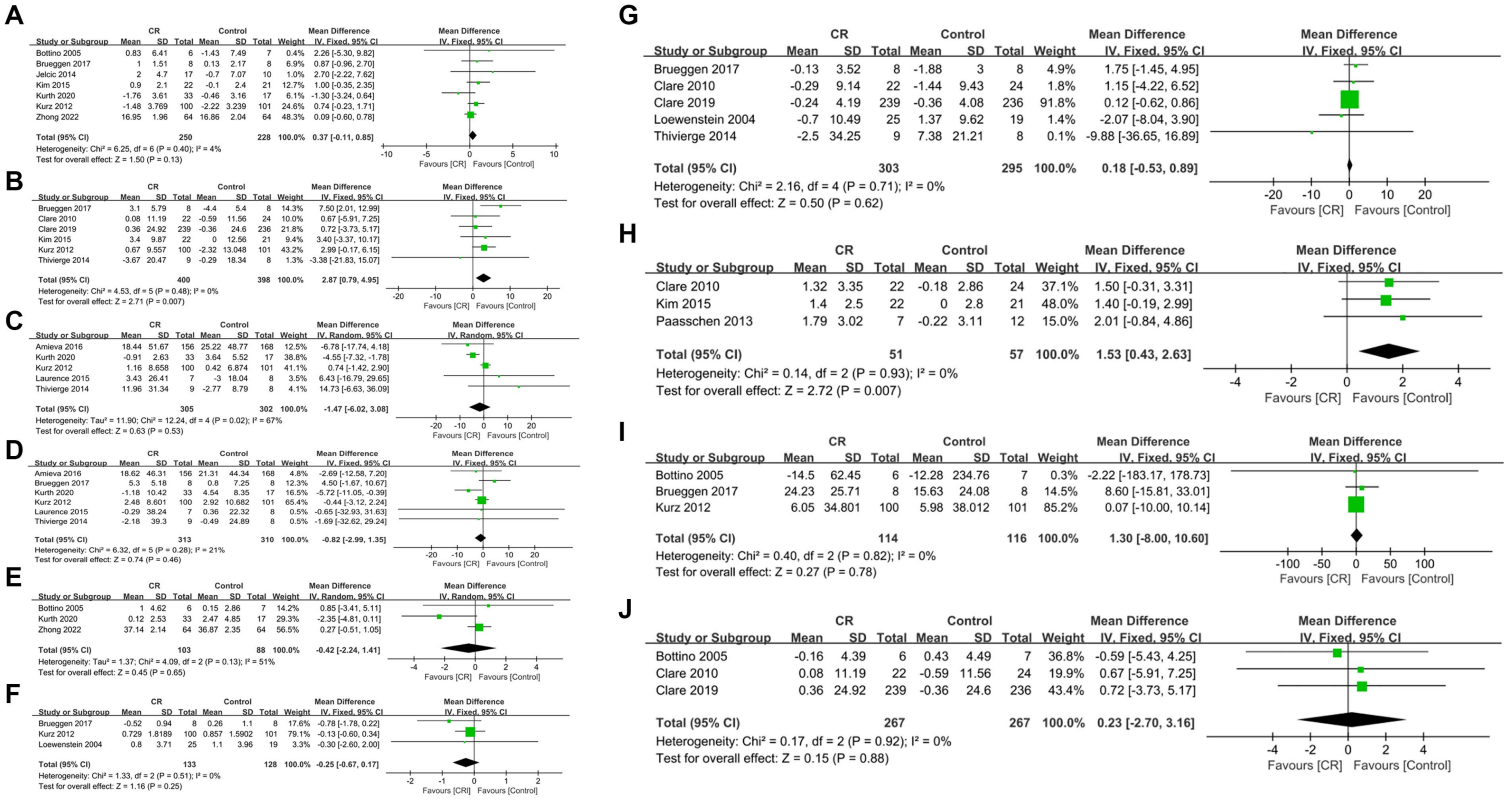
Forest plots of outcomes: **(A)** Mini-Mental State Examination, **(B)** Dementia quality of life, **(C)** neuropsychiatric inventory severity, **(D)** Zarit Burden interview, **(E)** activities of daily living, **(F)** Bayer activities of daily living, **(G)** Rivermead Behavioural memory test, **(H)** Canadian occupational performance measure, **(I)** trail marking test, **(J)** verbal fluency.

**Figure 4:**
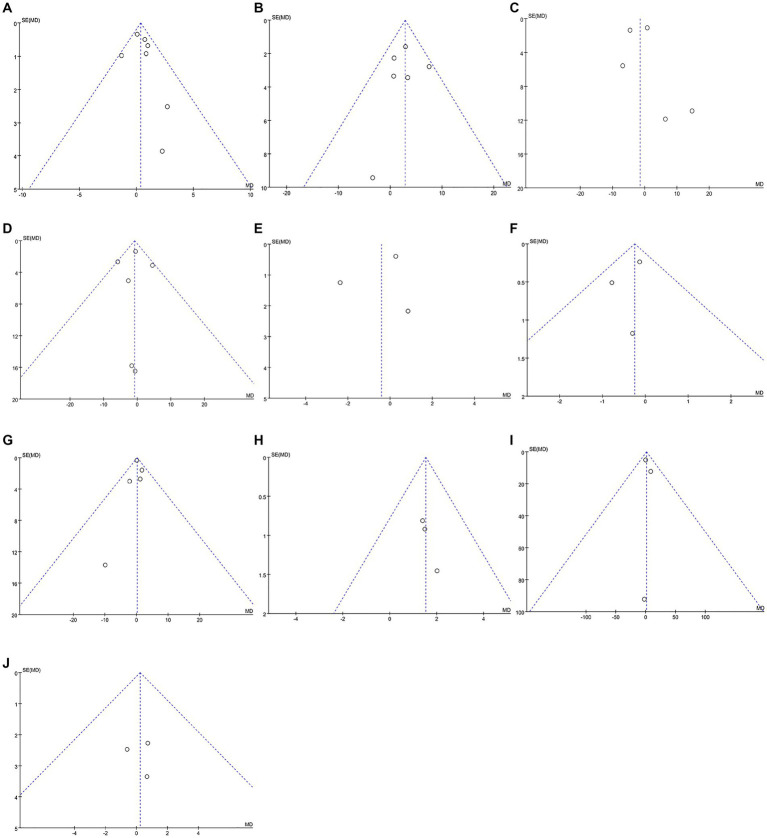
Funnel plots of outcomes: **(A)** Mini-Mental State Examination, **(B)** Dementia Quality of Life, **(C)** neuropsychiatric inventory severity, **(D)** Zarit Burden Interview, **(E)** activities of daily living, **(F)** Bayer activities of daily living, **(G)** Rivermead Behavioural memory test, **(H)** Canadian occupational performance measure, **(I)** trail marking test, **(J)** verbal fluency.

Data were obtained from seven studies involving 478 patients (250 cognitive rehabilitation patients vs. 228 control patients) (16, 19, 20, 22, 24–26). Pooled analyses showed no significant difference in MMSE between the two groups (WMD: 0.37; 95% CI: −0.11, 0.85; *p* = 0.13) and no significant heterogeneity was identified (*I*^2^ = 4%, *p* = 0.40) (Figure 3A). Additionally, publication bias was not observed either by the funnel plot (Figure 4A) or Egger’s test (*p* = 0.481).

#### 3.3.2 Change in DQoL

In DQoL analysis, six studies involving 798 patients (400 cognitive rehabilitation patients vs. 398 control patients) (18, 20, 24, 26–28) were included. Pooled analyses showed that cognitive rehabilitation significantly improved DQoL compared to the control group (WMD: 2.87; 95% CI: 0.79, 4.95; *p* = 0.007) (Figure 3B). Furthermore, no significant heterogeneity (*I*^2^ = 0%, *p* = 0.48) was identified. Moreover, publication bias was not detected either by the Egger test (*p* = 0.733) or visual evidence (Figure 4B).

#### 3.3.3 Change in NPI severity

The NPI severity analysis included five studies with 607 patients (305 cognitive rehabilitation patients vs. 302 control patients) (17–20, 29). Pooled results showed that no significant differences were detected between the two groups (WMD: −1.47; 95% CI: −6.02, 3.08; *p* = 0.53). However, the heterogeneity was detected (*I*^2^ = 67%, *p* = 0.02) (Figure 3C), with a slight publication bias (Figure 4C). Nonetheless, the results of the Egger test were not statistically significant (*p* = 0.852).

#### 3.3.4 Change in ZBI

Six articles with 623 patients (313 cognitive rehabilitation patients vs. 310 control patients) (17–20, 24, 29) reported ZBI. The results revealed similar ZBI between the two groups (WMD: −0.82; 95% CI: −2.99, 1.35; *p* = 0.46), and no significant heterogeneity was noticed (*I*^2^ = 21%, *p* = 0.28) (Figure 3D). No publication bias was detected by the funnel plot (Figure 4D) or Egger’s test (*p* = 0.901).

#### 3.3.5 Change in ADL

Three articles (comprising 103 cognitive rehabilitation patients and 88 control patients) reported data on ADL (16, 19, 22). The results showed similar ADL scores between the two groups (WMD: −0.42; 95% CI: −2.24, 1.41; *p* = 0.65), with no significant heterogeneity (*I*^2^ = 51%, *p* = 0.13) (Figure 3E). Additionally, publication bias was not observed either using Egger’s test (*p* = 0.670) or visual evidence (Figure 4E).

#### 3.3.6 Change in B-ADL

B-ADL was reported in three studies with 261 patients (133 cognitive rehabilitation patients vs. 128 control patients) (20, 21, 24). Pooled analyses revealed similar B-ADL between the two groups (WMD: −0.25; 95% CI: −0.67, 0.17; *p* = 0.25) with no significant heterogeneity (*I*^2^ = 0%, *p* = 0.51) (Figure 3F). No publication bias was identified by the funnel plot (Figure 4F) or the Egger test (*p* = 0.61).

#### 3.3.7 Change in RBMT

Data in the RBMT analysis was derived from five studies involving 598 patients (303 cognitive rehabilitation patients vs. 295 control patients) (18, 21, 24, 27, 28). The study showed no significant difference in RBMT between the two groups (WMD: 0.18; 95% CI: −0.53, 0.89; *p* = 0.62). Moreover, no significant heterogeneity (*I*^2^ = 0%, *p* = 0.71) (Figure 3G) and publication bias was not identified statistically (Egger’s test, *p* = 0.836) or visually (Figure 4G).

#### 3.3.8 Change in COPM

COPM analysis involved 108 patients (51 cognitive rehabilitation patients vs. 57 control patients) from three studies (23, 26, 28). The analysis demonstrated that patients in the cognitive rehabilitation group had considerably higher COPM scores than patients in the control group (WMD: 1.53; 95% CI: 0.43, 2.63; *p* = 0.007) (Figure 3E). Furthermore, no significant heterogeneity was observed (*I*^2^ = 0%, *p* = 0.93). No publication bias was detected in the funnel plot (Figure 4H), however, statistical evidence of publication bias was present (Egger’s test, *p* = 0.009).

#### 3.3.9 Change in TMTA

Three studies involving 230 patients (114 cognitive rehabilitation patients vs. 116 control patients) reported TMTA (20, 22, 24). The results exhibited similar TMTA scores between the two groups (WMD: 1.30; 95% CI: −8.00, 10.60; *p* = 0.78) with no significant heterogeneity (*I*^2^ = 0%, *p* = 0.82) (Figure 3I). Moreover, publication bias was not identified by either using the Egger test (*p* = 0.702) or visual (Figure 4I).

#### 3.3.10 Change in verbal fluency

Three articles provided data on verbal fluency for both two groups, comprising a total of 534 patients (267 cognitive rehabilitation patients vs. 267 control patients) (22, 27, 28). The analysis showed similar verbal fluency between the two groups (WMD: 0.23; 95% CI: −2.70, 3.16; *p* = 0.88) and no significant heterogeneity was observed (*I*^2^ = 0%, *p* = 0.92) (Figure 3J). Furthermore, publication bias was not detected by the funnel plot (Figure 4J) or Egger’s test (*p* = 0.909).

### 3.4 Sensitivity analyses

**Figure 5:**
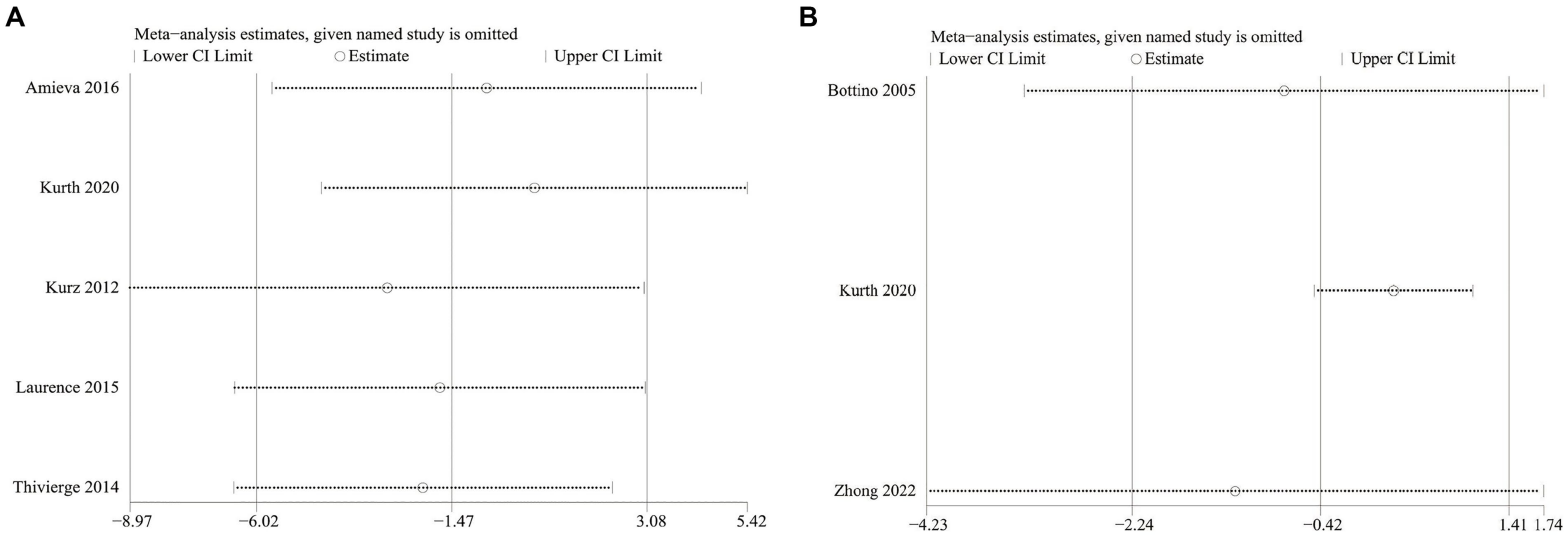
**(A)** Sensitivity analysis results of neuropsychiatric inventory severity; **(B)** sensitivity analysis results of activities of daily living.

We conducted one-way sensitivity analyses for NPI severity and ADL scores between the cognitive rehabilitation group and the control group. These analyses aimed to assess the influence of each study on the overall effect of combined WMD. The results of sensitivity analysis indicated that the new combined WMD was consistent after eliminating any individual studies for NPI severity (Figure 5A) and ADL (Figure 5B) scores. However, heterogeneity in NPI severity disappeared when we removed data reported by Kurth et al. in 2020 (*I*^2^ = 18%, *p* = 0.3) or Kurz et al. in 2012 (*I*^2^ = 27%, *p* = 0.25). Similarly, when we discarded data reported by Kurth et al. in 2020 (*I*^2^ = 0%, *p* = 0.79) or Zhong et al. in 2022 (*I*^2^ = 38%, *p* = 0.2), heterogeneity in ADL disappeared. This suggests that the exclusion of the two studies in each of the two outcome measures explains the heterogeneity.

### 3.5 Subgroup analyses

Subgroup analyses were conducted to explore the source of heterogeneity according to length of follow-up (greater than 6 months, less than or equal to 6 months) and regions (Asia, Europe, America) (Table 3). Pooled analyses showed similar DQoL between the two groups when follow-up was less than 6 months (WMD: 2.59; 95%CI: −3.76, 8.95; *p* = 0.42). However, when follow-up was longer than 6 months, DQoL in the cognitive rehabilitation group improved considerably compared to the control group (WMD: 2.90; 95%CI: 0.70, 5.10; *p* = 0.01). Importantly, no significant differences were observed between the two groups across various study regions.

**Table 3:** Subgroup analyses of outcomes.

Subgroup	Mini-Mental State Examination (MMSE)				Dementia Quality of Life (DQoL)				Neuropsychiatric Inventory severity (NPI severity)				Zarit Burden Interview (ZBI)			
	Study	MD [95%CI]	*p*-value	*I* ^2^	Study	MD [95%CI]	*p*-value	*I* ^2^	Study	MD [96%CI]	*p*-value	*I* ^2^	Study	MD [97%CI]	*p*-value	*I* ^2^
Total	7	0.37 [−0. 11,0.85]	0.13	4%	6	2.87 [0.79, 4.95]	0.007	0%	5	−1.47 [−6.02, 3.08]	0.53	67%	6	−0.82 [−2.99, 1.35]	0.46	21%
Follow-up																
>6 months	4	0.45 [−0.33, 1.23]	0.26	23%	4	2.90 [0.70, 5. 10]	0.01	26%	3	−2.48 [−7.14, 2.18]	0.3	79%	4	−0.82 [−2.99, 1.36]	0.46	52%
≤6 months	3	0.32 [−0.29, 0.93]	0.31	13%	2	2.59 [−3.76, 8.95]	0.42	0%	2	10.92 [−4.80, 26.65]	0.17	0%	2	−1.19 [−23.52, 21. 14]	0.92	0%
Region																
Asia	2	0.28 [−0.34, 0.90]	0.37	28%	1	3.40 [−3.37, 10. 17]	0.33	NA	0				0			
Europe	4	0.49 [−0.29, 1.26]	0.22	33%	4	2.90 [0.70, 5. 10]	0.01	26%	3	−2.48 [−7.14, 2.18]	0.3	79%	4	−0.82 [−2.99, 1.36]	0.46	52%
America	1	2.26 [−5.30, 9.82]	0.56	NA	1	−3.38 [−21.83, 15.07]	0.72	NA	2	10.92 [−4.80, 26.65]	0.17	0%	2	−1.19 [−23.52, 21. 14]	0.92	0%

Subgroup	Activities of daily living (ADL)				Bayer Activities of Daily Living (B-ADL)				Rivermead Behavioural Memory Test (RBMT)			
	Study	MD [97%CI]	*p*-value	*I* ^2^	Study	MD [97%CI]	*p*-value	*I* ^2^	Study	MD [97%CI]	*p*-value	*I* ^2^
Total	3	−0.42 [−2.24, 1.41]	0.65	51%	3	−0.25 [−0.67, 0.17]	0.25	0%	5	0.18 [−0.53, 0.89]	0.62	0%
Follow-up												
>6 months	2	−1.24 [−4.23, 1.74]	0.41	38%	3	−0.25 [−0.67, 0.17]	0.25	0%	4	0.19 [−0.53, 0.90]	0.61	0%
≤6 months	1	0.27 [−0.51, 1.05]	0.5	NA	0				1	−9.88 [−36.65, 16.89]	0.47	NA
Region												
Asia	1	0.27 [−0.51, 1.05]	0.5	NA	0				0			
Europe	1	−2.35 [−4.81, 0.11]	0.06	NA	2	−0.25 [−0.67, 0.18]	0.26	25%	3	0.22 [−0.50, 0.94]	0.55	0%
America	1	0.85 [−3.41, 5.11]	0.7	NA	1	−0.30 [−2.60, 2.00]	0.8	NA	2	−2.44 [−8.26, 3.39]	0.31	0%

Subgroup	Canadian Occupational Performance Measure (COPM)				Trail Marking Test (TMTA)				Verbal fluency			
	Study	MD [97%CI]	*p*-value	*I* ^2^	Study	MD [97%CI]	*p*-value	*I* ^2^	Study	MD [97%CI]	*p*-value	*I* ^2^
Total	3	1.53 [0.43, 2.63]	0.007	0%	3	1.30 [−8.00, 10.60]	0.78	0%	3	0.23 [−2.70, 3.16]	0.88	0%
Follow-up												
>6 months	1	1.50 [−0.31, 3.31]	0.1	NA	3	1.30 [−8.00, 10.60]	0.78	0%	3	0.23 [−2.70, 3.16]	0.88	0%
≤6 months	2	1.54 [0.16, 2.93]	0.03	0%	0				0			
Region												
Asia	1	1.40 [−0.19, 2.99]	0.08	NA	0				0			
Europe	2	1.65 [0.12, 3.17]	0.03	0%	2	1.31 [−8.00, 10.62]	0.78	0%	2	0.70 [−2.98, 4.39]	0.71	0%
America	0				1	−2.22 [−183.17, 178.73]	0.98	NA	1	−0.59 [−5.43, 4.25]	0.81	NA

Subgroup analysis for COPM, which reported in three studies, revealed that the COPM scores were greater in the cognitive rehabilitation group than in the control group when the follow-up period was less than 6 months (WMD: 1.54; 95% CI: 0.16, 2.93; *p* = 0.03), whereas the COPM scores of two groups were similar (WMD: 1.50; 95% CI: −0.31, 3.31; *p* = 0.1) when the follow-up period was longer than 6 months. In terms of the COPM scores, patients in the cognitive rehabilitation group had higher scores in Europe (WMD: 1.65; 95% CI: 0.12, 3.17; *p* = 0.03) than in Asia (WMD: 1.40; 95% CI: −0.19, 2.99; *p* = 0.08).

Moreover, four studies revealed similar NPI severity between the cognitive rehabilitation group and control group regardless of length of follow-up and regions. However, significant heterogeneity was observed at follow-up > 6 months (*I*^2^ = 79%) and in the European region (*I*^2^ = 79%), suggesting that follow-up > 6 months and studies conducted in the European region may contribute mainly to the high heterogeneity in the NPI scores. The ZBI scores were similar.

As for the other outcome indicators such as MMSE, ADL, B-ADL, NPI severity, ZBI, RBMT, TMTA and verbal fluency, no significant differences were identified between the cognitive rehabilitation group and the control group, with no significant heterogeneity (Table 3).

## 4 Discussion

AD leads to a decline in work or usual activities function, cognitive function, neuropsychiatric and self-care ability, which significantly reduces the patient well-being and imposes a heavy burden on families and society. AD shows several features such as progressive synapse damage, neuronal impairment, neuronal cell death, and vascular toxicity due to the accumulation of pathologically induced amyloid beta peptides and hyperphosphorylated tau protein in brain tissue (30). Current clinical research highlights the importance of improving brain tissue circulation and blood perfusion, as well as enhancing cognitive function by improving serum homocysteine levels, inflammatory factors, blood oxygen levels and other serological indicators in AD patients. However, pharmacological interventions aiming at combating these pathological changes have not consistently achieved lasting success. Moreover, pharmacological interventions can be costly and lead to various adverse effects. CR, on the other hand, is an individualized behavioral approach based on a problem-solving strategy and implements rehabilitation principles to ameliorate cognitive impairment. Given the nature and extent of cognitive impairment, CR is designed to enable individuals with cognitive impairment to perform at their highest potential, activating the brain by mediating neuroprotection, improving cortical connectivity, and changing brain morphology. This approach aims to reduce functional disability, improve the self-management ability of patients, as well as encourage them to engage in social activities (27).

This meta-analysis analyzed 14 clinical trials on the effects of cognitive rehabilitation on the overall cognitive functioning of people with AD (e.g., verbal fluency, behavioral memory, neuropsychiatric status, performing basic daily activities independently, and occupational performance levels). The study showed that CR exhibited a moderate to large impact on both quality of life (DQoL scores) (18, 20, 24, 26–28) and occupational performance levels (COPM scores) (23, 26, 28) in people with AD. In addition, the outcomes of the present study did not show significant effects of cognitive rehabilitation on specific cognitive functions domains of neuropsychiatric status, caregiver burden, independent performance on daily living, behavioral memory, attention, and verbal fluency in people with AD (16–22, 24–28).

### 4.1 Interpretation of results

This research demonstrates that CR is a promising approach in the treatment of AD. Instead of attempting to directly alter cognitive function, CR focuses on developing and implementing strategies in daily life, including external memory aids or daily routines, that are not demanding but can help individuals manage everyday challenges. CR addresses various methodological, psychological, and behavioral factors that are essential for maintaining daily functions, such as willpower, organization, judgment, planning, and sequencing. Patients are trained to use tailored strategies and apply new coping skills to meet their individualized daily needs (27). Studies have linked cognitive decline in AD patients to extensive structural brain abnormalities. Distinct patterns of memory dysfunction are correlated with particular patterns of gray matter loss, and the atrophy of medial temporal lobe may be a significant predictor of cognitive decline. In addition, relevant cortical regions responsible for the cognitive changes may also undergo changes in the plastic brain (31–33). Relevant clinical studies have examined the impact of CR on memory-related brain activation in AD patients using functional magnetic resonance imaging (fMRI). After receiving an 8 weeks personalized cognitive rehabilitation program intervention, the CR group showed greater brain activation in the left middle frontal gyrus, bilateral insula, and angular gyrus cortical areas (23). The CR group demonstrated enhanced connectivity in the angular cortex and superior frontal cortex, and increased connectivity over time in the supplementary motor cortex, postcentral gyrus, precuneus, insula, and paracentral lobule (34). Clare et al. discovered that patients in the CR group observed significant group-time interaction effects on both encoding and recognition when performing a face-name association task. Furthermore, patients in CR group demonstrated elevated Blood Oxygen level-dependent (BOLD) level (28), suggesting that AD patients can learn and adapt even with brain degeneration.

To the best of our knowledge, prior research on cognitive rehabilitation and AD is limited to systematic reviews. This is the first complete and systematic meta-analysis to unravel the effects of cognitive rehabilitation on the overall cognitive functioning of people with AD. Of 14 studies, six studies showed significant improvements in quality of life (18, 20, 24, 26–28) and three studies showed significant improvements in occupational performance levels (23, 26, 28). The findings suggest that cognitive rehabilitation could be an effective approach for improving cognitive functioning in people with AD, although its effects may be limited to specific cognitive functions domains.

### 4.2 Strengths and limitations

The advantage of this meta-analysis is that it includes both RCTs and non-RCTs, and most of the included studies are RCTs, which could provide more comprehensive data for analysis. At the same time, this study can quantitatively assess the overall impact and confirm the efficacy of cognitive rehabilitation on the overall cognitive function of AD patients. However, there are several limitations that should be taken into consideration when interpreting the current results. First, there is considerable heterogeneity observed among the included studies, particularly concerning the interventions used in the control group. The interventions and training measures used in the control group may not be sufficiently refined and precisely defined. Second, since this is a meta-analysis article, cognitive measurement may not cover all cognitive fields due to the inclusion of article data. This is unavoidable at the moment and requires more comprehensive and in-depth research in the future. Due to few studies (*N* = 14), it was not statistically appropriate to analyze the impact of different interventions compared to the cognitive rehabilitation group. However, a moderate analysis can still offer valuable insights for developing prevention strategies and designing appropriate interventions. Third, random assignment bias is prominent in the included studies, with some participants possibly selecting interventions that are more appropriate for them at the time of enrollment. This factor might have compromised the results. Finally, only studies reported in English are considered in the current meta-analysis, which potentially neglects some potentially eligible studies.

### 4.3 Implications for future research

Future research is required to gain extensive insights into the impact of each cognitive intervention, such as cognitive rehabilitation and cognitive training combination control, cognitive rehabilitation and cognitive stimulation combination control, in order to differentiate the role of the interventions in specific cognitive domains. It is particularly crucial to clarify the differences between cognitive rehabilitation, cognitive stimulation, and cognitive training and the direction of their respective adaptations. Given fewer studies in the field of cognitive rehabilitation for AD patients, there is a need for additional research endeavors to expand and improve the relevant research data. In addition, we suggest conducting more systematic and comprehensive controlled studies to assess the benefits of cognitive interventions. Moreover, the long-term effects of cognitive interventions should be examined, exploring the combination and ranking of various cognitive interventions to gain insights into possible maintenance effects. Future research is warranted to provide insights into the selection of appropriate cognitive intervention strategies.

Meanwhile, neuropsychiatric disorders commonly occur in the AD population, and are considered to be key risk factors for further cognitive decline and dementia. However, our study places less emphasis on this aspect. We searched for some newer related studies, such as the studies by Mokhtari et al. (35), Woolf et al. (36), and Motter et al. (35–37). These studies have systematically and deeply investigated cognitive rehabilitation or cognitive intervention for neuropsychiatric diseases. Their results show that cognitive rehabilitation or cognitive intervention has a moderate to significant impact on the cognition and emotion, daily executive functions, language learning, and working memory in MDD patients. Their conclusions are an important supplement to our study.

## 5 Conclusion

This study suggests that cognitive rehabilitation exhibited a moderate to large impact on both quality of life and occupational performance levels in people with AD. To further advance our understanding of the relative effectiveness of different cognitive intervention strategies within specific domains of functioning in patients with AD, it is essential to conduct well-designed randomized controlled trials on cognitive intervention strategies and with long-term follow-up. These endeavors will enable us to explore the advantages of different cognitive interventions and gain valuable insights into their beneficial effects in diverse domains of cognitive function.

## Data availability statement

The original contributions presented in the study are included in the article/Supplementary material, further inquiries can be directed to the corresponding author.

## Author contributions

SR: Conceptualization, Data curation, Investigation, Methodology, Writing – original draft, Writing – review & editing. FP: Conceptualization, Data curation, Investigation, Methodology, Writing – original draft. JJ: Conceptualization, Data curation, Formal analysis, Writing – original draft.
