# Supplementary material for: The effect of cognitive rehabilitation on daily functioning of patients with Alzheimer’s disease: a systematic review and meta-analysis of clinical trials
Source: Front Neurol. 2024 Apr 2;15:1371298. doi: 10.3389/fneur.2024.1371298 (PMC11019015; doi:10.3389/fneur.2024.1371298)
Supplement: Supplementary file 2 [file Table_2.DOCX]

**Appendix 2 Search strategy**

**Alzheimer Disease**

Alzheimer Dementia

Alzheimer Dementias

Dementia, Alzheimer

Alzheimer's Disease

Dementia, Senile

Senile Dementia

Dementia, Alzheimer Type

Alzheimer Type Dementia

Alzheimer-Type Dementia (ATD)

Alzheimer Type Dementia (ATD)

Dementia, Alzheimer-Type (ATD)

Alzheimer Type Senile Dementia

Primary Senile Degenerative Dementia

Dementia, Primary Senile Degenerative

Alzheimer Sclerosis

Sclerosis, Alzheimer

Alzheimer Syndrome

Alzheimer's Diseases

Alzheimer Diseases

Alzheimers Diseases

Senile Dementia, Alzheimer Type

Acute Confusional Senile Dementia

Senile Dementia, Acute Confusional

Dementia, Presenile

Presenile Dementia

Alzheimer Disease, Late Onset

Late Onset Alzheimer Disease

Alzheimer's Disease, Focal Onset

Focal Onset Alzheimer's Disease

Familial Alzheimer Disease (FAD)

Alzheimer Disease, Familial (FAD)

Familial Alzheimer Diseases (FAD)

Alzheimer Disease, Early Onset

Early Onset Alzheimer Disease

Presenile Alzheimer Dementia

**Pubmed-1243**

((("Alzheimer Disease"[Mesh]) OR (((((((((((((((((((((((((((((((((((Alzheimer Dementia) OR (Alzheimer Dementias)) OR (Dementia, Alzheimer)) OR (Alzheimer's Disease)) OR (Dementia, Senile)) OR (Senile Dementia)) OR (Dementia, Alzheimer Type)) OR (Alzheimer Type Dementia)) OR (Alzheimer-Type Dementia (ATD))) OR (Alzheimer Type Dementia (ATD))) OR (Dementia, Alzheimer-Type (ATD))) OR (Alzheimer Type Senile Dementia)) OR (Primary Senile Degenerative Dementia)) OR (Dementia, Primary Senile Degenerative)) OR (Alzheimer Sclerosis)) OR (Sclerosis, Alzheimer)) OR (Alzheimer Syndrome)) OR (Alzheimer's Diseases)) OR (Alzheimer Diseases)) OR (Alzheimers Diseases)) OR (Senile Dementia, Alzheimer Type)) OR (Acute Confusional Senile Dementia)) OR (Senile Dementia, Acute Confusional)) OR (Dementia, Presenile)) OR (Presenile Dementia)) OR (Alzheimer Disease, Late Onset)) OR (Late Onset Alzheimer Disease)) OR (Alzheimer's Disease, Focal Onset)) OR (Focal Onset Alzheimer's Disease)) OR (Familial Alzheimer Disease (FAD))) OR (Alzheimer Disease, Familial (FAD))) OR (Familial Alzheimer Diseases (FAD))) OR (Alzheimer Disease, Early Onset)) OR (Early Onset Alzheimer Disease)) OR (Presenile Alzheimer Dementia))) AND (cognitive rehabilitation)) AND (((((((randomized controlled trial) OR (RCT)) OR (cohort)) OR (clinical trial)) OR (clinical study)) OR (case-control)) OR (cross-sectional))

**Embase-249**


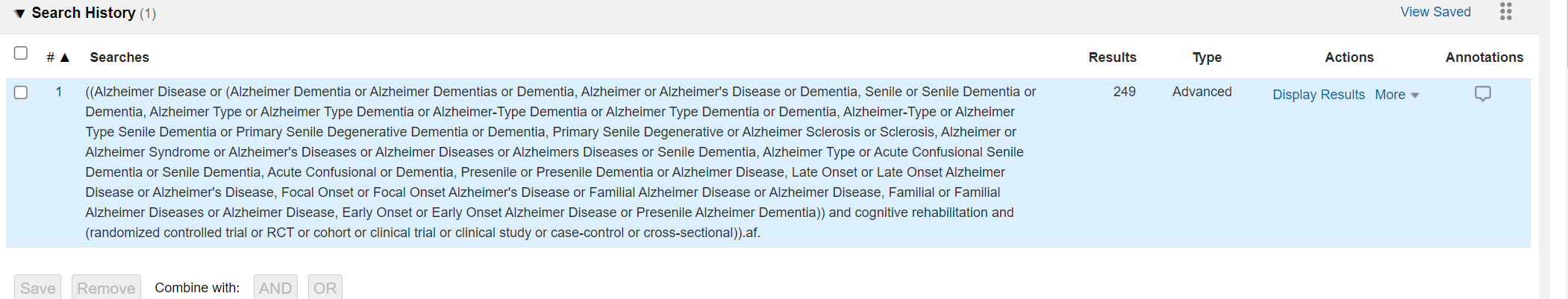


**Cochrane-69**


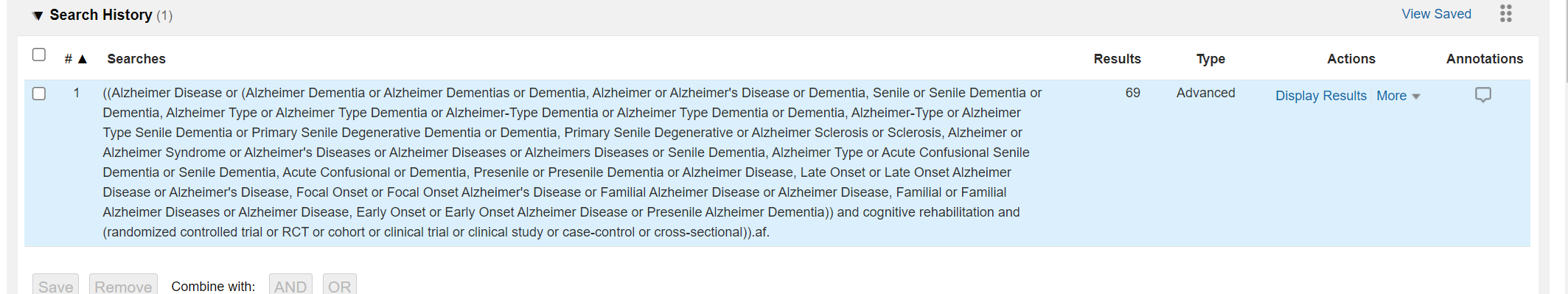


**Web of science-645**


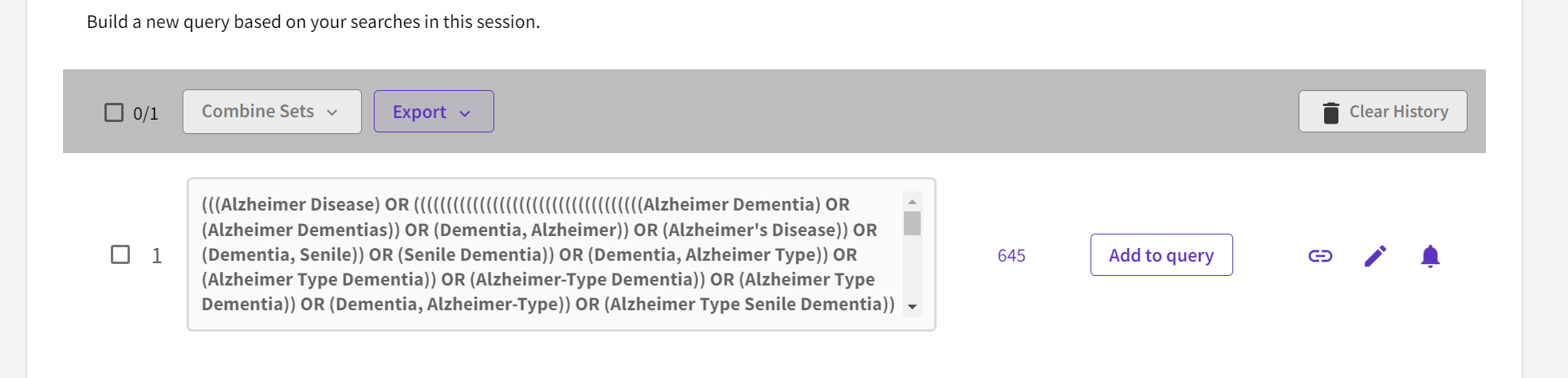


(((Alzheimer Disease) OR (((((((((((((((((((((((((((((((((((Alzheimer Dementia) OR (Alzheimer Dementias)) OR (Dementia, Alzheimer)) OR (Alzheimer's Disease)) OR (Dementia, Senile)) OR (Senile Dementia)) OR (Dementia, Alzheimer Type)) OR (Alzheimer Type Dementia)) OR (Alzheimer-Type Dementia)) OR (Alzheimer Type Dementia)) OR (Dementia, Alzheimer-Type)) OR (Alzheimer Type Senile Dementia)) OR (Primary Senile Degenerative Dementia)) OR (Dementia, Primary Senile Degenerative)) OR (Alzheimer Sclerosis)) OR (Sclerosis, Alzheimer)) OR (Alzheimer Syndrome)) OR (Alzheimer's Diseases)) OR (Alzheimer Diseases)) OR (Alzheimers Diseases)) OR (Senile Dementia, Alzheimer Type)) OR (Acute Confusional Senile Dementia)) OR (Senile Dementia, Acute Confusional)) OR (Dementia, Presenile)) OR (Presenile Dementia)) OR (Alzheimer Disease, Late Onset)) OR (Late Onset Alzheimer Disease)) OR (Alzheimer's Disease, Focal Onset)) OR (Focal Onset Alzheimer's Disease)) OR (Familial Alzheimer Disease)) OR (Alzheimer Disease, Familial)) OR (Familial Alzheimer Diseases)) OR (Alzheimer Disease, Early Onset)) OR (Early Onset Alzheimer Disease)) OR (Presenile Alzheimer Dementia))) AND (cognitive rehabilitation)) AND (((((((randomized controlled trial) OR (RCT)) OR (cohort)) OR (clinical trial)) OR (clinical study)) OR (case-control)) OR (cross-sectional))
